# Deep learning assisted contrast-enhanced CT–based diagnosis of cervical lymph node metastasis of oral cancer: a retrospective study of 1466 cases

**DOI:** 10.1007/s00330-022-09355-5

**Published:** 2022-12-28

**Authors:** Xiaoshuai Xu, Linlin Xi, Lili Wei, Luping Wu, Yuming Xu, Bailve Liu, Bo Li, Ke Liu, Gaigai Hou, Hao Lin, Zhe Shao, Kehua Su, Zhengjun Shang

**Affiliations:** 1 The State Key Laboratory Breeding Base of Basic Science of Stomatology (Hubei-MOST) & Key Laboratory of Oral Biomedicine Ministry of Education, School & Hospital of Stomatology, Wuhan University, Wuhan, China; 2 School of Computer Science, Wuhan University, 299 Bayi Road, Wuhan, 430072 Hubei China; 3 Department of Radiology, School & Hospital of Stomatology, Wuhan University, Wuhan, China; 4 Department of Oral and Maxillofacial Head Neck Surgery, School & Hospital of Stomatology, Wuhan University, 237 Luoyu Road, Wuhan, 430079 Hubei China

**Keywords:** Mouth neoplasms, Lymphatic metastasis, Tomography, X-ray computed, Deep learning, Diagnosis, computer-assisted

## Abstract

**Objectives:**

Lymph node (LN) metastasis is a common cause of recurrence in oral cancer; however, the accuracy of distinguishing positive and negative LNs is not ideal. Here, we aimed to develop a deep learning model that can identify, locate, and distinguish LNs in contrast-enhanced CT (CECT) images with a higher accuracy.

**Methods:**

The preoperative CECT images and corresponding postoperative pathological diagnoses of 1466 patients with oral cancer from our hospital were retrospectively collected. In stage I, full-layer images (five common anatomical structures) were labeled; in stage II, negative and positive LNs were separately labeled. The stage I model was innovatively employed for stage II training to improve accuracy with the idea of transfer learning (TL). The Mask R-CNN instance segmentation framework was selected for model construction and training. The accuracy of the model was compared with that of human observers.

**Results:**

A total of 5412 images and 5601 images were labeled in stage I and II, respectively. The stage I model achieved an excellent segmentation effect in the test set (AP_50_-0.7249). The positive LN accuracy of the stage II TL model was similar to that of the radiologist and much higher than that of the surgeons and students (0.7042 vs. 0.7647 (*p* = 0.243), 0.4216 (*p* < 0.001), and 0.3629 (*p* < 0.001)). The clinical accuracy of the model was highest (0.8509 vs. 0.8000, 0.5500, 0.4500, and 0.6658 of the Radiology Department).

**Conclusions:**

The model was constructed using a deep neural network and had high accuracy in LN localization and metastasis discrimination, which could contribute to accurate diagnosis and customized treatment planning.

**Key Points:**

*• Lymph node metastasis is not well recognized with modern medical imaging tools.*

*• Transfer learning can improve the accuracy of deep learning model prediction.*

*• Deep learning can aid the accurate identification of lymph node metastasis.*

**Supplementary Information:**

The online version contains supplementary material available at 10.1007/s00330-022-09355-5.

## Introduction

Oral cancer is a prevalent malignancy worldwide with a high recurrence rate [1, 2]. Lymph node (LN) metastasis is a common cause of recurrence for oral cancer patients [3]. Poor identification of LN metastasis often causes undertreatment with occult metastasis (30–40%) and overtreatment at an early stage (60–70%), both of which could cause poor prognosis [4, 5]. Additionally, whether elective neck dissection should be performed and the extent of tissue removal essentially depend on the diagnosis of LN metastasis.

Currently, CT and MRI are frequently utilized to identify the size, internal heterogeneity, and contour of LNs [6, 7]. With the adjunction of [^18^F]FDG PET, these imaging approaches can reach higher sensitivity [8, 9]. Despite advancements in modern medical imaging technology, even experienced radiologists often miss diagnoses with LN metastasis due to limited working time and a heavy daily workload [10, 11]. Additional invasive procedures, such as fine-needle aspiration biopsy, could be performed to increase the diagnostic accuracy of suspicious LNs, which is harmful to patients [12]. Therefore, more accurate and noninvasive LN metastasis diagnosis methods are needed.

As a subdomain of machine learning (ML), deep learning (DL) is a method that uses more complex network model structures, is much better at discovering deeper features in input data, and performs well in many practical application scenes of ML [13, 14]. Many studies have shown that DL achieves excellent performance in image processing problems [15–17]. DL-based image classification and object detection have been widely employed in the medical field to provide supporting advice for diagnoses [13, 18–24], such as skin cancer [25], breast cancer (LNs) [26], and COVID-19 [27]. Furthermore, DL can achieve or exceed the performance of human experts in several tasks of medical image analysis [28, 29]. In the field of medical image processing based on DL, the lack of annotated datasets is a major problem [18]. The introduction of transfer learning (TL) to DL can alleviate the shortage of annotated datasets. TL can improve the performance of learning on target domains by transferring knowledge from different but related source domains [30].

To help decrease the high misdiagnosis rate of LNs, it is worthwhile to introduce artificial intelligence (AI) to the field of imaging identification of LNs in oral cancer. Consequently, this research intends to develop a DL model that can identify, locate, and distinguish LNs in CECT images with a higher accuracy to replace existing inefficient manual identification methods.

## Materials and methods

### Patient cohort

This study was reported according to the recommendations of the STROBE guidelines and was approved by the Institutional Review Board (IRB) of the Ethics Committee of the Hospital of Stomatology, Wuhan University (IRB No. 2020-B63). As it was a retrospective study, all patients could not be identified, only imaging data and pathological reports were collected, and the extracted data did not contain patient names. Therefore, consent for participation was not obtained.

**Fig. 1:**
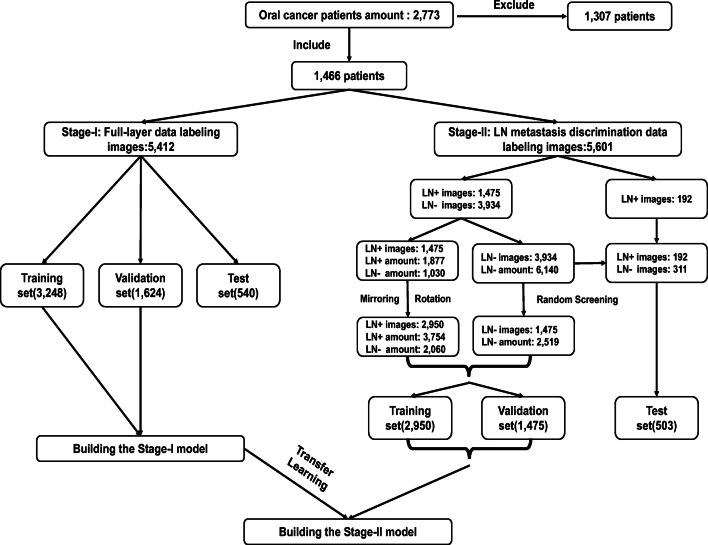
Process of patient data collection, screening, classification, and data labeling. Flowchart of the study population and assignments for date labeling and machine learning for construction of prediction models. LN, lymph node; LN+, positive lymph node; LN−, negative lymph node

The CECT images of 2773 patients with oral cancer who were admitted to the Hospital of Stomatology, Wuhan University, between September 1, 2012, and September 22, 2020, were retrospectively collected. After screening according to our inclusion and exclusion criteria, 1307 invalid samples were removed (Table S1). The baseline information of the included patients is shown in Table 1 and Table S2. For the 1466 included samples, 5412 images were selected for full-layer data labeling (stage I), and 5601 images were selected for LN metastasis discrimination data labeling (stage II) (Fig. 1).

**Table 1:** Baseline information of 1466 oral cancer patients in this study

		Total (*N* = 1466)	Male (*N* = 1059)	Female (*N* = 407)
Age (median [IQR])		55.41 [48,64]	54.80 [48,63]	57.01 [49,67]
T (tumor stage)	T1	34 (2.32%)	20 (1.36%)	14 (0.96%)
	T2	434 (29.60%)	287 (19.58%)	147 (10.02%)
	T3	693 (47.27%)	532 (36.29%)	161 (10.98%)
	T4a	305 (20.81%)	220 (15.01%)	85 (5.80%)
	T4b	0	0	0
N (node stage)	N0	778 (53.07%)	559 (38.13%)	219 (14.94%)
	N1	268 (18.28%)	189 (12.89%)	79 (5.39%)
	N2a	15 (1.02%)	10 (0.68%)	5 (0.34%)
	N2b	304 (20.74%)	225 (15.35%)	79 (5.39%)
	N2c	23 (1.57%)	17 (1.16%)	6 (0.41%)
	N3a	0	0	0
	N3b	78 (5.32%)	59 (4.02%)	19 (1.30%)
M (metastasis stage)	M0	1466 (100%)	1059 (72.24%)	407 (27.76%)
	M1	0	0	0
Clinical stage	I	29 (1.98%)	18 (1.23%)	11 (0.75%)
	II	310 (21.15%)	205 (13.99%)	105 (7.16%)
	III	511 (34.85%)	385 (26.26%)	126 (8.59%)
	IVA	537 (36.63%)	392 (26.74%)	145 (9.89%)
	IVB	79 (5.39%)	59 (4.03%)	20 (1.36%)
	IVC	0	0	0

We used the Joint Commission on Cancer eighth edition TNM classification (TNM8) criteria

*IQR* interquartile range

### Data processing

Three researchers were specially trained by a radiologist with 20 years of working experience in our hospital to be responsible for this study. All data were labeled based on pathological information for consensus regarding segmentation accuracy. If a consensus was not reached, researchers consulted the radiologist to reduce personnel errors. After the labeling was completed, it was reviewed and approved (or modified as needed) by the radiologist.

**Fig. 2:**
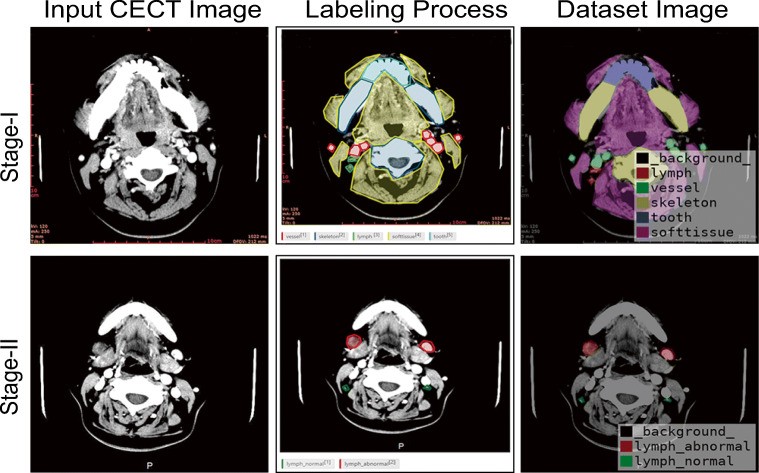
The process of data labeling. The process of data labeling for the input CECT images of stage I and stage II using our data-labeling tools. The dataset image used for training was generated from labeled data

This study was carried out in two stages. In stage I, CECT images were screened according to the traditional CT level guidelines for cervical LNs (seven categories) [31]. Most of the possible locations of LNs were included in the images, especially the most common levels, I to V. The target areas were outlined by forming polygons based on Label Studio (a privately deployed platform, which is based on the open-source data-labeling platform). For example, LNs were outlined by polygons consisting of green dots and lines. The remaining structures that appeared in the image were synchronously outlined, such as teeth (light blue), bone (dark blue), blood vessels (red), and other soft tissue (yellow). In stage II, positive LN (LN+) and negative LN (LN−) were outlined by polygons consisting of red (LN+) and green (LN−) dots and lines, respectively, based on Label Studio (Fig. 2). Detailed information on data processing (including CT parameters) is provided in the Supplement.

### Model introduction

Mask R-CNN, a simple, flexible, and general DL framework in the field of instance segmentation, was introduced in this study. Mask R-CNN adds a branch of mask generation based on Faster R-CNN, which can realize object detection and generate high-quality masks for different instances of an object [32].

The relevant code based on the Mask R-CNN framework using TensorFlow was implemented. The network structure consisted of three parts: the backbone network, region proposal network (RPN), and head network (Fig. S1 and S2).

Due to the similar nature of the training tasks in these two stages, both of which were multiclass instance segmentation (five classes in stage I and two classes in stage II), the overall network structure was similar, but the training process parameter settings were different.

ResNet101, which is composed of 101 layers, was selected as the backbone feature extraction network, and simultaneously constructed feature pyramids based on a feature pyramid network (FPN). In the following part, C1 to C5 were employed to define the feature map output by different ResNet convolutional layers [16]. The construction of the FPN was to realize the fusion of features at multiple scales [33]. The extracted P2 to P6 could be utilized as the effective feature layer of the RPN (Fig. S2).

The RPN was used to generate a region of interest (RoI) for the head network [34]. Considering the size of the input image, five anchors of different scales—16 × 16, 32 × 32, 64 × 64, 128 × 128, and 256 × 256—were defined. The gridlines were generated through the RPN area, and the preselection bounding boxes that may contain objects were generated and referred to as the proposal area. A set of RoIs was generated according to the evaluation of each proposal area, which greatly reduced the task size of subsequent classification detection.

The head network, which included a classifier model and mask generation model, was responsible for classification and mask generation. Both branches were simultaneously employed. The head network determined the object category of the input RoI through a classifier model and a generated mask for each instance of the object by classifying the pixel level through a mask generation model [32].

To minimize the influence of overfitting, the L2 regularization method was utilized for the model.

### Model training

In the two stages, the loss function (*L*) applied was multitask loss, which was the sum of classification loss (*L*_cls_), bounding-box loss (*L*_box_), and mask branch loss (*L*_mask_).

$$ L={L}_{\mathrm{cls}}+{L}_{\mathrm{box}}+{L}_{\mathrm{mask}} $$

In our study, the datasets were shuffled and split into training (60%), validation (30%), and test sets (10%). For model training, Mask R-CNN COCO model weights were used as the initial weights, which were optimized by the stochastic gradient descent (SGD) strategy.

In stage I, the model was trained for 50 epochs in total on the training set and validated after each epoch on the validation set. The trends of loss during training and validation are shown in Fig. S3a, b.

In stage II, the LN metastasis discrimination labeled data (enlarged by means of rotation and mirroring) were served as the dataset. The stage II model (without TL) was trained for 100 epochs in total. The trends of loss are shown in Fig. S3c, d. Subsequently, the stage I model of the training results was used as the initial model weight for TL to improve the effect. However, the stage II-TL model (with TL) was trained for 10 epochs in total. The trends of loss are shown in Fig. S3e, f.

During training, validation loss was calculated after each epoch, and model weights were saved following each epoch that showed improvement in validation loss.

### Assessment by clinicians

After model construction and optimization, another radiologist with 11 years of working experience, two surgeons with 12 and 11 years of experience, and two graduate students with 3 years of clinical experience in the Department of Oral and Maxillofacial Head Neck Surgery were invited to identify and distinguish LNs+. The accuracy of the model was compared with clinicians’ results to assess the clinical usability of the model.

### Statistical analysis

The chi-square test was performed to examine the differences in the categorical variables. A two-sided *p* < 0.05 means that the corresponding estimate reaches a significant difference. The performance of the stage I model was evaluated on the independent test set using the average precision (AP) of the model to measure the segmentation effect. However, in stage II, the AP could not directly reflect the overall effect of the model. Hence, three new customized but much stricter model evaluation criteria were introduced: LN accuracy, LN+ accuracy, and clinical accuracy. Detailed information about the calculation of the AP and PR curves and these three new evaluation criteria are provided in the Supplement.

## Results

### Identify different anatomical structures (stage I)

**Fig. 3:**
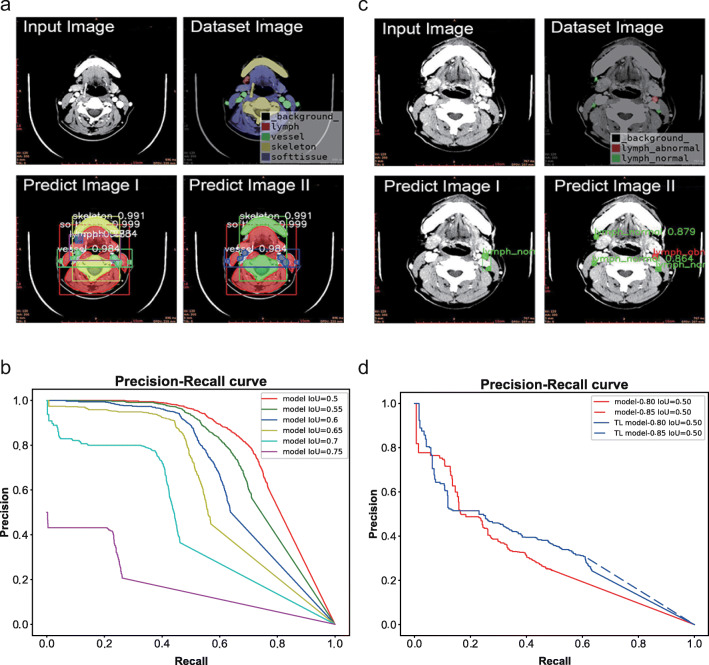
Prediction process and PR curves. **a** Prediction process using the stage I model. Predicted images I and II were generated from the input image with confidence levels of 0.75 and 0.85, respectively. The dataset image was labeled for comparison. **b** PR curves at different IoU threshold values in stage I. **c** Prediction process using the stage II model. Predicted image I was generated from the input image in the stage II model. Predicted image II was generated from the input image in the stage II-TL model. The dataset image was labeled for comparison. **d** PR curves of the model before and after the stage I model was utilized for training of transfer learning at the default IoU threshold value (0.50)

Stage I aimed to automatically recognize different soft and hard tissues in the cervical CECT images, especially to distinguish and identify LNs, hoping to achieve more accurate recognition and positioning of LNs in the next stage of training. All anatomical structures, including teeth, bones, LNs, blood vessels, and other soft tissues were labeled (Fig. 2). A total of 5412 CECT images were screened. The prediction result image and precision/recall (PR) curve for different intersection-over-union (IoU) thresholds of the stage I model are shown in Fig. 3a, b.

AP_50_, AP_60_, AP_70_, and AP reached 72.49%, 58.72%, 35.88%, and 29.63%, respectively, for the discriminant segmentation of different anatomical structures in the test set (Table 2). The AP rapidly decreased with an increase in the IoU threshold. Considering the small size of LNs and blood vessels in images, the number of negative samples increased with an increase in the IoU threshold. In summary, in stage I, the different anatomical structures could be effectively recognized in the cervical CECT images.

**Table 2:** AP of each model at different IoU thresholds

	AP	AP_50_	AP_60_	AP_70_
Stage I model	29.63	72.49	58.72	35.88
Stage II model-0.80	\	24.35	\	\
Stage II-TL model-0.80	\	30.63	\	\
Stage II model-0.85	\	24.35	\	\
Stage II-TL model-0.85	\	29.97	\	\

*AP* average precision, *TL* transfer learning,"\" means there is no value

### Accuracy of cervical LN metastasis discrimination was improved by TL (stage II)

Stage II was designed to accurately distinguish LN metastasis. LN− and LN+ were specifically labeled (Fig. 2). A total of 5601 CECT images were screened, in which 2126 were LNs+, with 1667 images involved, and 6140 were LNs−, with 3934 images involved (Fig. 1).

Since AP_50_ represented the best recognition effect in the stage I model, only AP_50_ was shown in different stage II models (Table 2). The prediction result images and the PR curve at the default IoU (0.50) threshold of the stage II model are shown in Fig. 3c, d.

However, only the partial neck levels that underwent elective neck dissection had LNs’ pathological information for the labeling process, while the LNs’ pathological information in the neck levels without surgery was ambiguous, and the LN status in these levels could not be labeled. But the prediction included levels without pathological information, and when these predicted instance objects appeared in the model evaluation, the corresponding instance could not be identified in the labeled samples. As a result, many negative samples were generated during the matching calculation, which affected the overall evaluation of the model, and its AP could not directly reflect the effect of the model. Hence, three new customized but much stricter model evaluation criteria were introduced: LN accuracy, LN+ accuracy, and clinical accuracy.

Notably, when the stage I model was employed in stage II for TL, the stage II-TL model significantly improved the discrimination of cervical LN metastasis (Tables 3, 4 and 5) (*p* < 0.001). Each indicator reached the highest value at the 0.85 confidence level (Table 5). In conclusion, the stage II-TL model achieved the highest LN accuracy (71.90%), LN+ accuracy (70.42%), and clinical accuracy (85.09%) at the 0.85 confidence level.

**Table 3:** Chi-square test between TL model-0.80 and model-0.80

	Right	Wrong	Accuracy (%)	*p* values
TL model-0.80	209	135	60.76	
Model-0.80	179	272	39.69	*p* = 3.92274E−09

**Table 4:** Chi-square test between TL model-0.85 and other models or clinicians

	Right	Wrong	Accuracy (%)	*p* values
TL model-0.85	200	84	70.42	
Model-0.85	175	253	40.89	*p* = 1.08402E−14
TL model-0.80	209	135	60.76	*p* = 0.011407545
TL model-0.84	202	94	68.24	*p* = 0.569445805
TL model-0.86	198	84	70.21	*p* = 0.956442618
TL model-0.90	158	108	59.40	*p* = 0.006720646
Radiologist	78	24	76.47	*p* = 0.243141608
Surgeons	85	119	42.16	*p* = 2.05967E−10
Students	90	159	36.29	*p* = 2.23635E−15

**Table 5:** Stage II predictions of each model and predictions of radiologist, surgeons, students, and the Radiology Department

	Clinical accuracy	LN accuracy	Positive LN accuracy			
			Total	Level I	Level II	Level III
Model-0.80	58.45 (294/503)	45.62 (583/(1007 + 271))	39.69 (179/(249 + 202))	33.63	46.63	33.68
TL model-0.80	76.34 (384/503)	71.76 (846/(1007 + 172))	60.76 (209/(249 + 95))	56.60	64.38	53.62
Model-0.85	60.44 (304/503)	42.33 (527/(1007 + 238))	40.89 (175/(249 + 179))	34.93	46.24	36.36
TL model-0.85	85.09 (428/503)	71.90 (801/(1007 + 102))	70.42 (200/(249 + 35))	66.67	72.22	65.45
TL model-0.84	83.10 (418/503)	71.83 (811/(1007 + 122))	68.24 (202/(249 + 47))	64.18	70.00	62.07
TL model-0.86	85.09 (428/503)	68.38 (757/(1007 + 100))	70.21 (198/(249 + 33))	65.60	72.00	66.06
TL model-0.90	82.31 (414/503)	53.01 (563/(1007 + 55))	59.40 (158/(249 + 17))	51.69	60.17	55.88
Radiologist	80.00 (80/100)	\	76.47 (78/(91 + 11))	\	\	\
Surgeons	55.00 (55/100)	\	42.16 (43/(91 + 11))	\	\	\
Students	45.00 (45/100)	\	36.29 (45/(91 + 33))	\	\	\
Radiology Department	66.58 (976/1466)	\	\	\	\	\

*TL* transfer learning, *LN* lymph node,"\"means there is no value

### Comparison of predicting accuracy in different neck levels

**Fig. 4:**
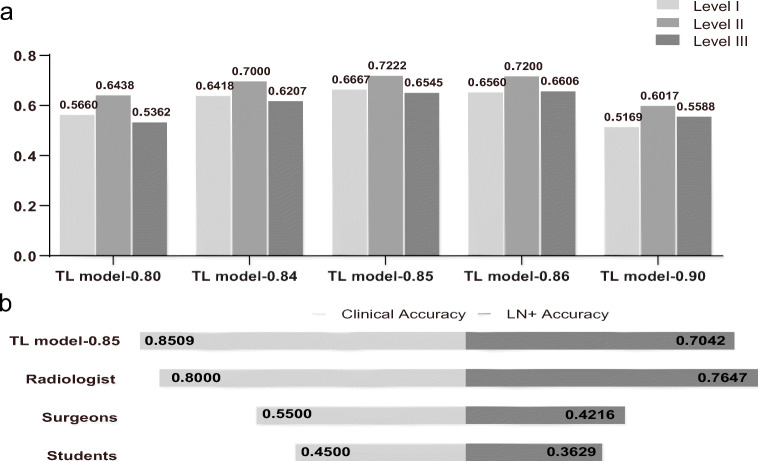
Model evaluation and prediction results. **a** LN+ accuracy of the stage II-TL model at different levels at different confidence intervals. **b** Comparison results of LN+ accuracy and clinical accuracy among the stage II-TL model-0.85, radiologist, surgeons, and students. TL, transfer learning; LN+, positive lymph node

The neck is divided into a total of seven levels based on the topographical subdivision [30, 31]. In the stage II test set, the data at levels I, II, and III were dominant, with 195, 191, and 198 images, respectively. These three levels were peculiarly prone to LN metastasis in oral cancer. Therefore, the prediction results of LN+ accuracy at these levels were compared. The results indicated that for different models and different confidence levels, the highest LN+ accuracy was obtained at level II (Fig. 4a and Table 5).

### Prediction of cervical LN metastasis: model versus clinicians

To verify the accuracy of the model, the stage II-TL model-0.85 predictions were compared with those of the radiologist, surgeons, surgical students, and diagnostic reports from the Radiology Department in our hospital. Considering that the accurate identification of LN+ is the most important indicator, LN+ accuracy and clinical accuracy were selected for the comparison of the prediction results. Unfortunately, medical imaging diagnostic reports did not describe every LN, so the LN+ accuracy and LN accuracy of the Radiology Department could not be calculated.

Collectively, our model achieved LN+ accuracy, which was comparable to that of the radiologist (70.42% vs. 76.47%) (*p* = 0.243), and its LN+ accuracy was much higher than that of the surgeons (42.16%) (*p* < 0.001) and students (36.29%) (*p* < 0.001) (Table 4). Our model achieved the highest clinical accuracy (85.09% vs. 80%, 55%, 45%, and 66.58% of the Radiology Department) (Fig. 4b and Table 5).

## Discussion

In this study, DL using a neural network combined with CECT images could identify, locate, and distinguish cervical metastatic LNs of oral cancer patients with a very high accuracy and was demonstrated to exceed that of an experienced radiologist and surgeons. Even in terms of clinical accuracy, our model exceeded the average of the Radiology Department in our hospital by nearly 20%. To the best of our knowledge, this is the largest study on the prediction of cervical LN metastasis by CECT images based on DL in oral cancer. Our model was very effective not only in assisting radiologists in accurately diagnosing LN metastasis before surgery but also in helping surgeons in designing customized and accurate surgical plans to improve prognosis.

For many years, neck dissection has been a major concern for oral cancer patients worldwide. With all fields being facilitated by AI, especially in the medical imaging field, it is regrettable that few studies have carried out AI analysis on cervical LNs [25, 27, 35]. Reza et al and Yuan et al performed texture analysis on cervical LN metastasis by dual-energy CT and MRI, respectively, based on ML with high accuracy [36, 37]. The content of our study was CECT, consistent with China’s national conditions because of their strong universality and applicability. [^18^F]FDG PET/CT can better detect occult neck metastasis than CT/MRI imaging [9]. However, this method is expensive, and only a few large hospitals are equipped with such expensive machines. Additionally, the sample sizes adopted by previous researchers were measured in units of 10 and 100, while the present study directly collected data from 2773 patients and included 1466 samples after screening. Most significantly, the present study first divided the work into two stages and innovatively integrated the idea of TL to apply the results of the first stage of the model to the training of the second stage of the model, which significantly improved the prediction accuracy. In previous studies, the prediction required an experienced operator to outline the LNs before the model recognized them, which was laborious and time-consuming. In this study, we need only to input a complete CECT image set of the patient into the model, after which the prediction results can be obtained in seconds, which is convenient and time-efficient with strong clinical translation ability.

As the model built by combining DL and radiology was noninvasive, we could use only CECT image to perform operational analysis before surgery and to minimize harm to the patients. Although the results of our study were impressive and provide a basis for future research, more challenging tasks, such as the identification of LNs at different levels, need to be completed [31]. Nearly all patients included in the study had neck LN dissection at levels I, II, and III (images dominated the dataset), while only some patients had surgery at levels IV and V. Notably, levels I to III were peculiarly prone to LN metastasis in oral cancer. Incorrect discrimination of LNs at levels I to III is prone to increase as LNs at these levels are often accompanied by blood vessels. In the statistical results of our study, we presented the first analysis of discrepancies in the accuracy of the prediction of LNs at different levels and discovered that level II (upper group of the internal jugular chain) had a much higher accuracy at any confidence level in any model.

Although the AI utilized in the present study could greatly facilitate the diagnosis of diseases by clinicians, the current study has limitations. First, this was a single-center retrospective experiment; in particular, the LNs in pathological reports and CECT images were not completely accurate in one-to-one correspondence. Second, persuading patients to embrace this new diagnostic system remains challenging and requires large-scale clinical application and verification. Third, because the current ability of medical imaging (e.g., CT and MRI) to detect micrometastases in LNs with normal morphology and dimension is rather limited [6], our model, which is based on CECT imaging, still cannot reach satisfactory prediction within this aspect. Last, multicenter prospective studies and a larger sample size are needed to validate our results to popularize our AI results and to overcome the subtle discrepancies of different CECT machines. Given these issues, AI has great potential in cervical LN metastasis diagnosis; however, whether it can be successfully promoted remains to be determined.

In conclusion, despite the fact that more framework optimization and large-scale verification are still required before official clinical application, AI-DL-Mask R-CNN–assisted CECT can accurately predict the metastasis of cervical LNs, providing strong support for the efficient diagnosis of radiologists to a certain extent. Importantly, convenient manipulation, instant diagnosis, and excellent effect endow this technology with a powerful clinical translation ability.

## Supplementary information

ESM 1 (DOCX 330 kb)

## Abbreviations

AI: Artificial intelligence
AP: Average precision
CECT: Contrast-enhanced computed tomography
DL: Deep learning
Faster R-CNN: Faster region–based convolutional neural network
FPN: Feature pyramid network
IoU: Intersection over union
IRB: Institutional review board
LN: Lymph node
LN−: Negative LN
LN+: Positive LN
Mask R-CNN: Mask region–based convolutional neural network
ML: Machine learning
PR: Precision/recall
ResNet101: Residual Network101
RoI: Region of interest
RPN: Region proposal network
SGD: Stochastic gradient descent
TL: Transfer learning

## Declarations

### Guarantor

The scientific guarantor of this publication is Zhengjun Shang.

### Statistics and biometry

No complex statistical methods were necessary for this paper.

### Methodology

• retrospective

• diagnostic or prognostic study

• performed at one institution
