# Supplementary material for: Deep learning assisted contrast-enhanced CT–based diagnosis of cervical lymph node metastasis of oral cancer: a retrospective study of 1466 cases
Source: Eur Radiol. 2022 Dec 28;33(6):4303–12. doi: 10.1007/s00330-022-09355-5 (PMC9795159; doi:10.1007/s00330-022-09355-5)
Supplement: Supplementary file 1 — (DOCX 330 kb) [file 330_2022_9355_MOESM1_ESM.docx]

**Supplementary data**

1. **Data processing**

CECT was performed using a 16-slice spiral CT machine (GE, BrightSpeed). CECT scans were of diagnostic quality, using 120 kVp energy, a slice thickness of 5 mm, and iodinated contrast administered intravenously. All the data involved in this study were downloaded in DICOM format in their original dimensions and resolution and eventually processed and stored in the JPG format.

To ensure a sufficient number of positive samples for evaluation, 192 LN+ images were selected from 1,667 LN+ images and added to the test set, while the remaining 1,475 images were used for training and verification. Considering that uneven data distribution between LN+ and LN- samples would affect the applicability of the network model, we preprocessed the dataset. First, for the 1,475 LN+ images used for training and verification, mirroring, rotating, and other methods were randomly used to generate new samples to expand the data of the LN+ set. A total of 1,475 LN- images were randomly selected from 3,934 LN- images and added to the dataset for training and validation. The total number of extended training and validation datasets was 4,425, containing 3,754 LNs+ and 4,579 LNs-. Then 311 LN- images randomly selected from the remaining 2,459 LN- images and the previously reserved 192 LN+ images made up of the test set, which contained 249 LNs+ and 758 LNs-. The test set was isolated and blinded from view until final testing.

To have enough LNs+ to identify and ensure that the prediction and comparison effect was statistically significant, we randomly extracted 67 LN+ images and 33 LN- images, for a total of 100 CECT images, containing 91 LNs+ and 131 LNs-.

**2. AP and PR curve**

AP was selected to measure the segmentation effect for the instance segmentation model. The AP result calculated according to the default mask Intersection over Union (IoU) threshold value of 0.50 is represented as AP_50_. AP_60_ and AP_70_ were named in the same way. At the same time, when the IoU threshold was set every 0.05 increments from 0.50 to 0.95, a group of 10 APs were calculated, and the average crossing was taken to represent AP. Mask IoU is the ratio of intersection and union between the mask of the instance object in the prediction result and the mask of the corresponding instance object in the annotation.

$$IoU=\frac{Area of Overlap}{Area of Union}$$

The AP value represents the area of the region formed by the precision/recall (PR) curve and the coordinate axes. Precision represents the proportion of correctly predicted instances to all positively predicted instances at a given IoU threshold. Recall represents the proportion of correctly predicted instances to all true positive instances at a given IoU threshold.

The average precision at a set of 11 equally spaced recall levels [0, 0.1..., 1] is defined as AP [1]:

$$AP=\frac{1}{11}\sum_{r\in\left\{ 0,0.1,...,1 \right\}}^{n} P_{interp}(r)$$

For each recall level r, precisions are counted in which the corresponding recall exceeds r, and the maximum precision is used for interpolation to generate the measured precision [1]:

$$P_{\mathrm{interp}}(r)=\max_{r:\bar{r}>r} p(\tilde{r})$$

where p(r˜) is the measured precision at recall r˜ [1].

**3. Three new evaluation criteria**

In Stage-II, only the partial neck levels that underwent elective neck dissection had pathological information of LNs for the labeling process in the CECT images, while the pathological information of LNs in the neck levels without surgery was not clear, and the LN status in these levels could not be labeled. However, the prediction of the Stage-II model included levels without pathological information indication, and when these predicted instance objects appeared in the model evaluation, the corresponding instance could not be found in the labeled samples. As a result, many negative samples were generated during the matching calculation, which affected the overall evaluation of the model, and its AP could not directly reflect the overall effect of the model. Hence, three new customized but much stricter model evaluation criteria were introduced: LN Accuracy, LN+ Accuracy and Clinical Accuracy.

The LN instances with accurate location and correct feature discrimination were determined as correct instance T, and the LN+ instances with accurate location and correct feature discrimination were determined as correct instance PT. In the level indicated by pathological information, the incorrect discrimination or location of LN instances was determined as false instance F, and PF was named in the same way. The sum of all LN instances in the pathological information indicated level was S, and PS was named in the same way.

LN Accuracy refers to the ratio of the number of LN correctly predicted to all LNs (including labeled LNs and incorrect discrimination or location LNs) in the test set.

$$LN Accuracy=\frac{T}{S+F}$$

LN+ Accuracy refers to the ratio of the number of LN+ correctly predicted to all LNs+ (including labeled LNs+ and incorrect discrimination or location LNs+) in test set.

$$(LN+) Accuracy=\frac{\mathrm{PT}}{PS+PF}$$

In addition, Clinical Accuracy means that all LNs+ in the sample image are correctly identified without missing or incorrect discrimination. Clinical Accuracy was raised because the final goal of this study was to be applied in the clinic and assist surgeons in developing customized treatment plans.

1 Everingham M, Van Gool L, Williams CKI, Winn J, Zisserman A (2010) The Pascal Visual Object Classes (VOC) Challenge. International Journal of Computer Vision 88:303-338

**Figure legends**

Figure S1. Network architecture for training. It consists of three parts, backbone network, region proposal network and head network. ResNet101, residual network101; FPN, feature pyramid network; RoI, region of interest.


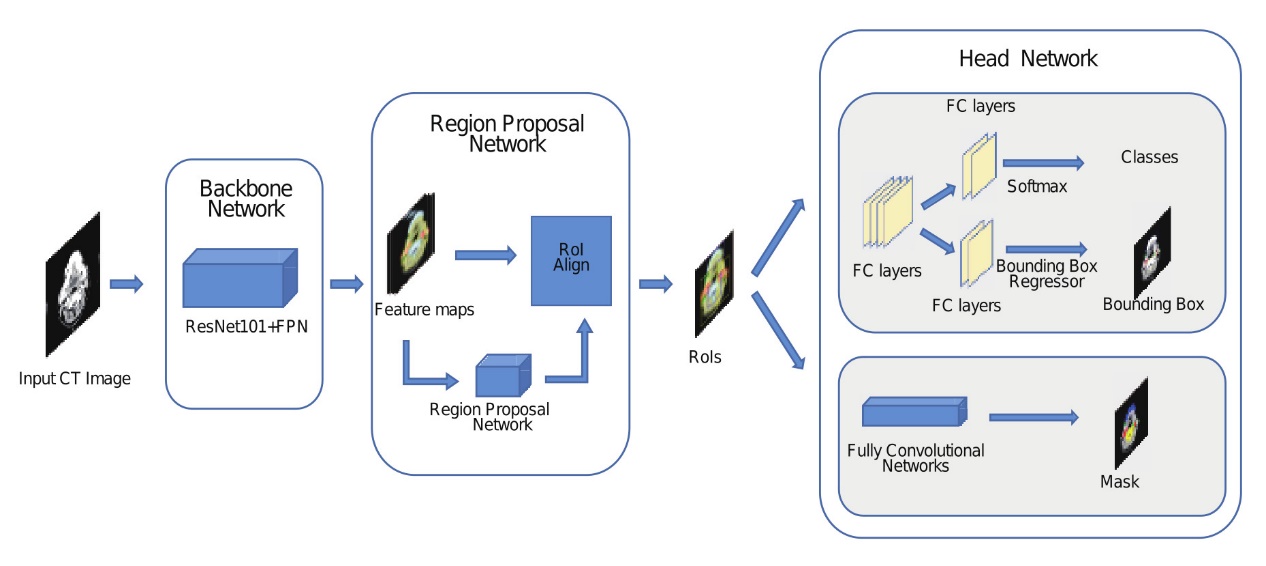


Figure S2. Feature fusion process. Firstly, C2, C3, C4 and C5 were convolved with 1×1, and P5 was obtained after operation of C5. 67 Then, P4 was obtained by UpSampling of P5 and C4 convolved with 1×1. P2 and P3 were generated as the same. In order to be 68 consistent with the Scale dimension in the RPN, P6 is obtained by MaxPooling through P5. RPN, region proposal network.


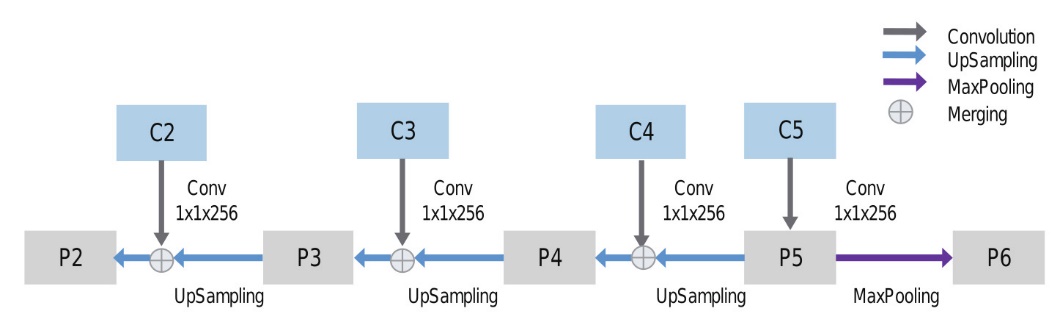


Figure S3. Loss curve during training and validation. Loss curve during training (a) and validation (b) at Stage-I of 50 epochs. Loss curve during training (c) and validation (d) at Stage-II of 100 epochs. Loss curve during training (e) and validation (f) at Stage-II-TL of 10 epochs.TL, transfer learning.


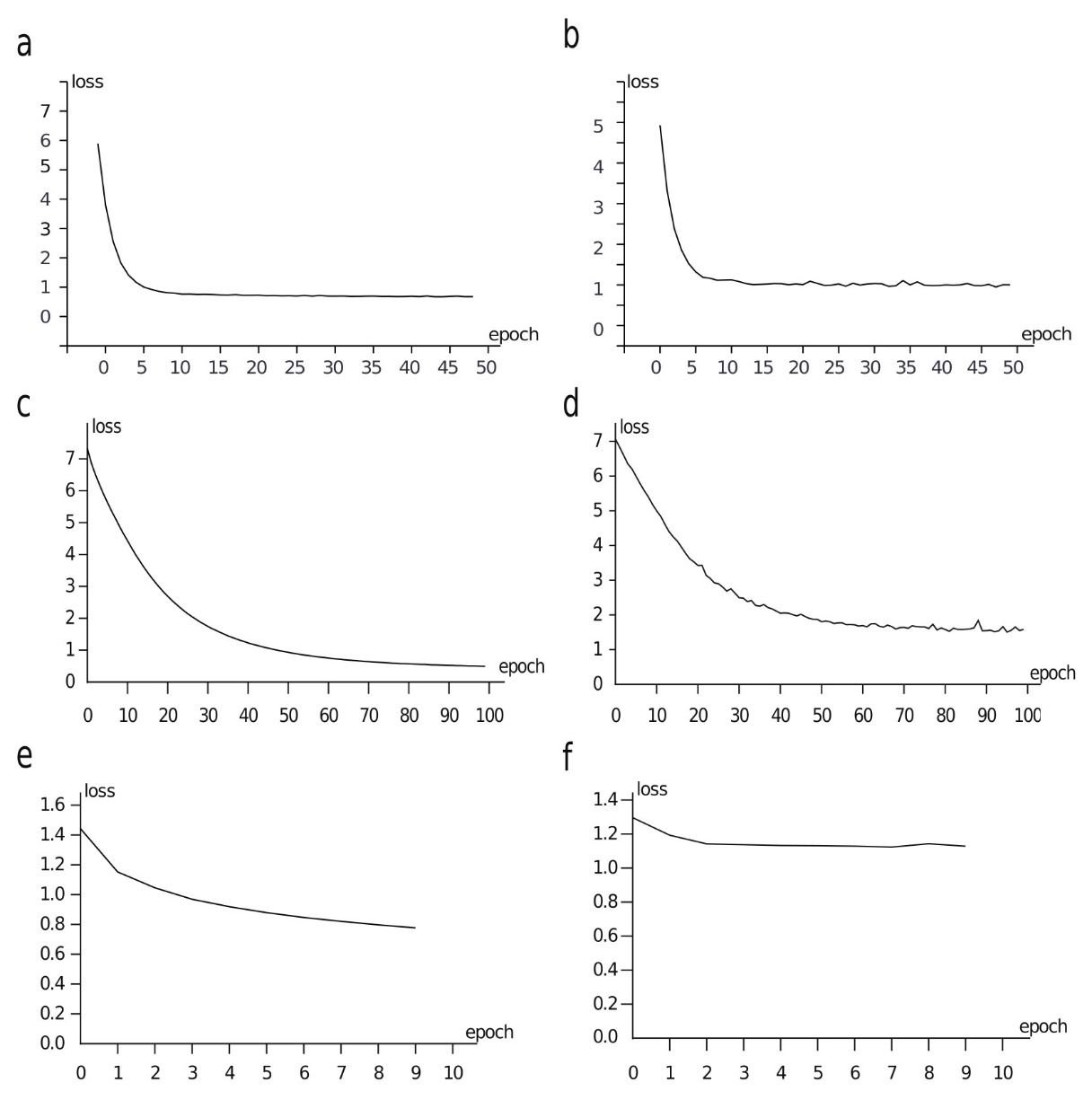


**Supplementary Table**

**Table S1.** Inclusion and exclusion criteria for patient selection.

| Inclusion criteria： | Exclusion criteria： | Additional exclusions： |
| --- | --- | --- |
| Pathologically confirmed oral cancer with one or more positive (metastatic) lymph nodes by elective neck dissection (688).  Pathologically confirmed oral cancer with negative (non-metastatic) lymph nodes by elective neck dissection (778). | All lymph nodes <5mm or not clearly visualized on CT slice (6).  Metastatic oral cancer lymph nodes: radiologic and pathological relevance is uncertain (17).  Lymph nodes cannot be reliably identified (2).  Lymph nodes metastatic disease without an identifiable primary tumor (3). | No enhanced image (462).  No 5 mm layer (685).  No pathological information (102).  No clear and valid image (13).  Patients not in the Department of Oral and Maxillofacial Head Neck Surgery (11).  Surgery was not performed within one month of CT photography (6). |

**Table S2.** Patient’s tumor site and primary/ recurrent information.

|  | **Total(N=1,466)** | **Primary(N=1,256)** | **Recurrent (N=210)** |
| --- | --- | --- | --- |
| Buccal | 309(21.08%) | 277(18.90%) | 32(2.18%) |
| Floor of mouth | 246(16.78%) | 213(14.53%) | 33(2.25%) |
| Hard palate | 78(5.32%) | 67(4.57%) | 11(0.75%) |
| Lip | 5(0.34%) | 5(0.34%) | 0 |
| Tongue | 601(41.00%) | 510(34.79%) | 91(6.21%) |
| Retromolar trigone and soft palate | 111(7.57%) | 96(6.55%) | 15(1.02%) |
| Gingiva and alveolar | 116(7.91%) | 88(6.00%) | 28(1.91%) |
